# Deficiency in the double-stranded RNA binding protein HYPONASTIC LEAVES1 increases sensitivity to the endoplasmic reticulum stress inducer tunicamycin in Arabidopsis

**DOI:** 10.1186/s13104-019-4623-3

**Published:** 2019-09-14

**Authors:** Rikako Hirata, Kei-ichiro Mishiba, Nozomu Koizumi, Yuji Iwata

**Affiliations:** Graduate School of Life and Environmental Sciences, Osaka Prefecture University, 1-1 Gakuen-cho, Naka-ku, Sakai, 599-8531 Osaka Japan

**Keywords:** *Arabidopsis thaliana*, Endoplasmic reticulum, HYPONASTIC LEAVES1, microRNA, Unfolded protein response

## Abstract

**Objective:**

microRNA (miRNA) is a small non-coding RNA that regulates gene expression by sequence-dependent binding to protein-coding mRNA in eukaryotic cells. In plants, miRNA plays important roles in a plethora of physiological processes, including abiotic and biotic stress responses. The present study was conducted to investigate whether miRNA-mediated regulation is important for the endoplasmic reticulum (ER) stress response in Arabidopsis.

**Results:**

We found that *hyl1* mutant plants are more sensitive to tunicamycin, an inhibitor of *N*-linked glycosylation that causes ER stress than wild-type plants. Other miRNA-related mutants, *se* and *ago1*, exhibited similar sensitivity to the wild-type, indicating that the hypersensitive phenotype is attributable to the loss-of-function of HYL1, rather than deficiency in general miRNA biogenesis and function. However, the transcriptional response of select ER stress-responsive genes in *hyl1* mutant plants was indistinguishable from that of wild-type plants, suggesting that the loss-of-function of HYL1 does not affect the ER stress signaling pathways.

## Introduction

microRNA (miRNA) is a non-coding small RNA that regulates gene expression in eukaryotic cells. miRNA binds to mRNA through nucleotide complementarity and mediates mRNA degradation and translation inhibition. The molecular mechanism of miRNA biogenesis has been extensively characterized in both animal and plant systems. In plants, miRNA is transcribed as a precursor RNA, termed primary miRNA (pri-miRNA) and processed by DICER-LIKE1 (DCL1) to generate a miRNA/miRNA* duplex [1]. One of the strands, miRNA, is incorporated into ARGONAUTE1 (AGO1) to form RNA-induced silencing complex (RISC) to target mRNA to mediate mRNA degradation and translation repression whereas the other strand, miRNA*, is degraded. A number of proteins, such as the double-stranded RNA (dsRNA) binding protein HYPONASTIC LEAVES1 (HYL1) and the zinc finger protein SERRATE (SE), have been reported to function in ensuring accurate and efficient miRNA biogenesis [2–4].

In plants, miRNA plays pivotal roles in a plethora of physiological processes, including abiotic and biotic stress responses and nutrient adaptation [5]. Indeed, a number of stress-related miRNAs have been identified in various plant species. For instance, Arabidopsis has been reported to accumulate miRNAs in response to salinity, drought, and cold [6]. Another example is that a salt-tolerant and a salt-sensitive line of maize express differential miRNA accumulation profiles [7]. miR399, miR395, and miR398 have been shown to be important for phosphate, sulfate, and copper homeostasis in plants [5].

The endoplasmic reticulum (ER) is the site of synthesis and maturation of secretory and membrane proteins in eukaryotic cells. ER stress occurs when protein folding and assembly in the ER is perturbed and unfolded and misfolded proteins accumulate in the ER. ER stress triggers a cellular response to maintain homeostasis called the ER stress response or the unfolded protein response (UPR) [8, 9]. IRE1 is an ER stress sensor widely conserved in eukaryotes. IRE1 is a type-I membrane protein and harbors the ER luminal sensor domain and the cytosolic kinase and ribonuclease domains [10]. In plants, IRE1 mediates transcriptional activation of genes encoding ER chaperones and folding enzymes, including BINDING PROTEIN (BiP), through cytoplasmic splicing of *bZIP60* mRNA, which produces an active transcription factor [11, 12]. IRE1 also mediates degradation of mRNA encoding secretory and membrane proteins to reduce the load of newly synthesized proteins into the stressed ER [13].

Although regulation of gene expression by miRNA is involved in a number of stress responses in plants, its involvement in the ER stress response has not been investigated. The present study was conducted to investigate a possible involvement of miRNA-related proteins in the ER stress response in Arabidopsis.

## Main text

### Methods

#### Plant materials

We used *Arabidopsis thaliana* Col-0 as wild-type and previously reported mutants in Col-0 background, *hyl1-2* [4], *se-1* [14], *ago1-46* [15], and *ire1ab* [11]. HYL1-YFP/*hyl1-2* is *hyl1-2* mutant expressing HYL1-YFP fusion proteins under *HYL1* promoter [16].

#### Sensitivity assay to ER stress-inducing agents

The sensitivity assay was carried out as previously described [17]. Briefly, Arabidopsis seeds of indicated genotypes were surface sterilized and sown on half-strength Murashige Skoog (MS) medium containing 1% sucrose supplemented with indicated concentrations of tunicamycin or 0.1% dimethyl sulfoxide (DMSO) as a solvent control. Sterilized seeds were grown under 16 h-light/8 h-dark cycle at 23 °C for 10 days and photographed. Seedlings with open cotyledons were counted as survived. Error bars represent standard errors calculated from three biological replicates.

#### Quantitative reverse transcription-PCR (qRT-PCR)

Ten-day-old wild-type and mutant seedlings grown in half-strength MS medium containing 1% sucrose were treated with 5 μg/mL tunicamycin for indicated time periods and ground by using a mortar and pestle with liquid nitrogen. Total RNA was extracted using acid guanidinium thiocyanate, phenol, and chloroform as described elsewhere [18].

One hundred ng RNA was reverse transcribed by High-Capacity cDNA Reverse Transcription Kit (Applied Biosystems) using random hexamer according to the manufacturer’s instruction. Quantitative real-time PCR was performed by using Applied Biosystems 7300 Real-Time PCR Systems (Applied Biosystems) with THUNDERBIRD SYBR qPCR Mix (TOYOBO) according to the manufacturer’s instruction. Primers used were listed in Additional file 1. Error bars represent standard errors calculated from three biological replicates.

### Results

#### hyl1 mutant is more sensitive to ER stress

**Fig. 1:**
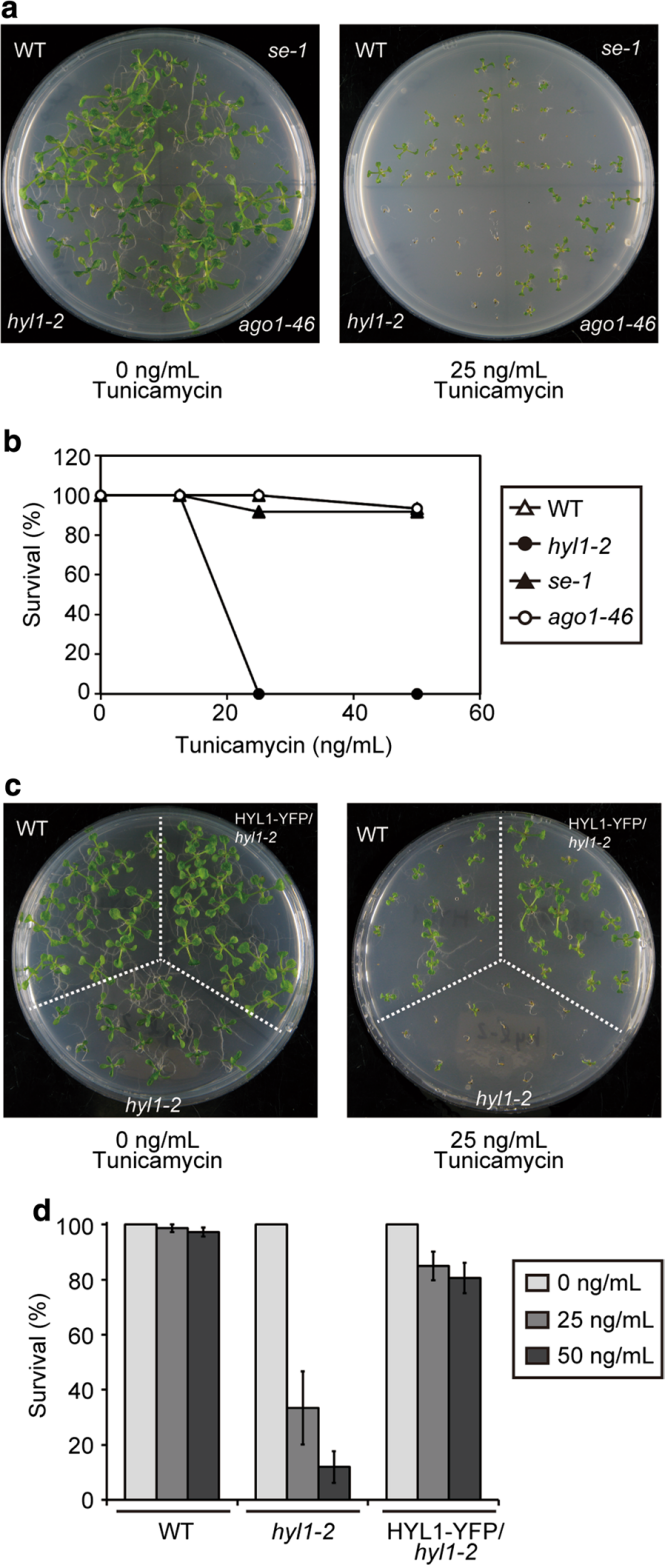
Sensitivity of Arabidopsis miRNA-related mutants to ER stress. **a** Sensitivity of miRNA-related mutants to tunicamycin. Seeds of wild-type (WT), *hyl1-2*, *se-1*, and *ago1-46* were sown on half-strength MS medium with indicated concentrations of tunicamycin, grown for 10 days, and photographed. **b** Effect of tunicamycin on survival of miRNA-related mutants. Seeds of indicated genotypes were grown for 10 days as in **a**, and the percentage of seeds that survived was calculated. **c** Sensitivity of *hyl1-2* and HYL1-YFP/*hyl1-2* plants to tunicamycin. Seedlings of wild-type (WT), *hyl1-2*, and HYL1-YFP/*hyl1-2* were grown as in **a** and photographed. **d** Effect of tunicamycin on survival of *hyl1-2* and HYL1-YFP/*hyl1-2* plants. Seeds of indicated genotypes were grown for 10 days as in **c**, and the percentage of seeds that survived was calculated

We first asked whether miRNA-related Arabidopsis mutants exhibit altered ER stress sensitivity. We examined *hyl1-2*, *se-1*, and *ago1-46* mutants for ER stress sensitivity. As shown in Fig. 1a, compared to wild-type seedlings, growth of *hyl1-2* mutant seedlings was more severely affected by treatment with tunicamycin, an inhibitor of *N*-linked glycosylation, which disrupts folding of glycosylated proteins, thereby causing ER stress. In contrast, growth of *se-1* and *ago1-46* mutant seedlings was similar to that of the wild-type seedlings. As shown in Fig. 1b, the survival rate was also significantly affected in *hyl1-2* mutant, but not in *se-1* and *ago1-46* mutants, when grown on tunicamycin-containing MS medium. The observation that only *hyl1-2* mutant was more sensitive to tunicamycin suggests that the hypersensitive phenotype of *hyl1-2* mutant to tunicamycin is attributed to the deficiency in the function of HYL1, rather than the defective miRNA biogenesis.

This hypersensitive phenotype of the *hyl1-2* mutant was complemented by expressing the *HYL1-YFP* fusion construct (Fig. 1c, d), confirming that the disruption of *HYL1* accounts for the observed tunicamycin hypersensitivity.

#### A transcriptional response of hyl1 mutant plants appears to be indistinguishable from that of wild-type plants

**Fig. 2:**
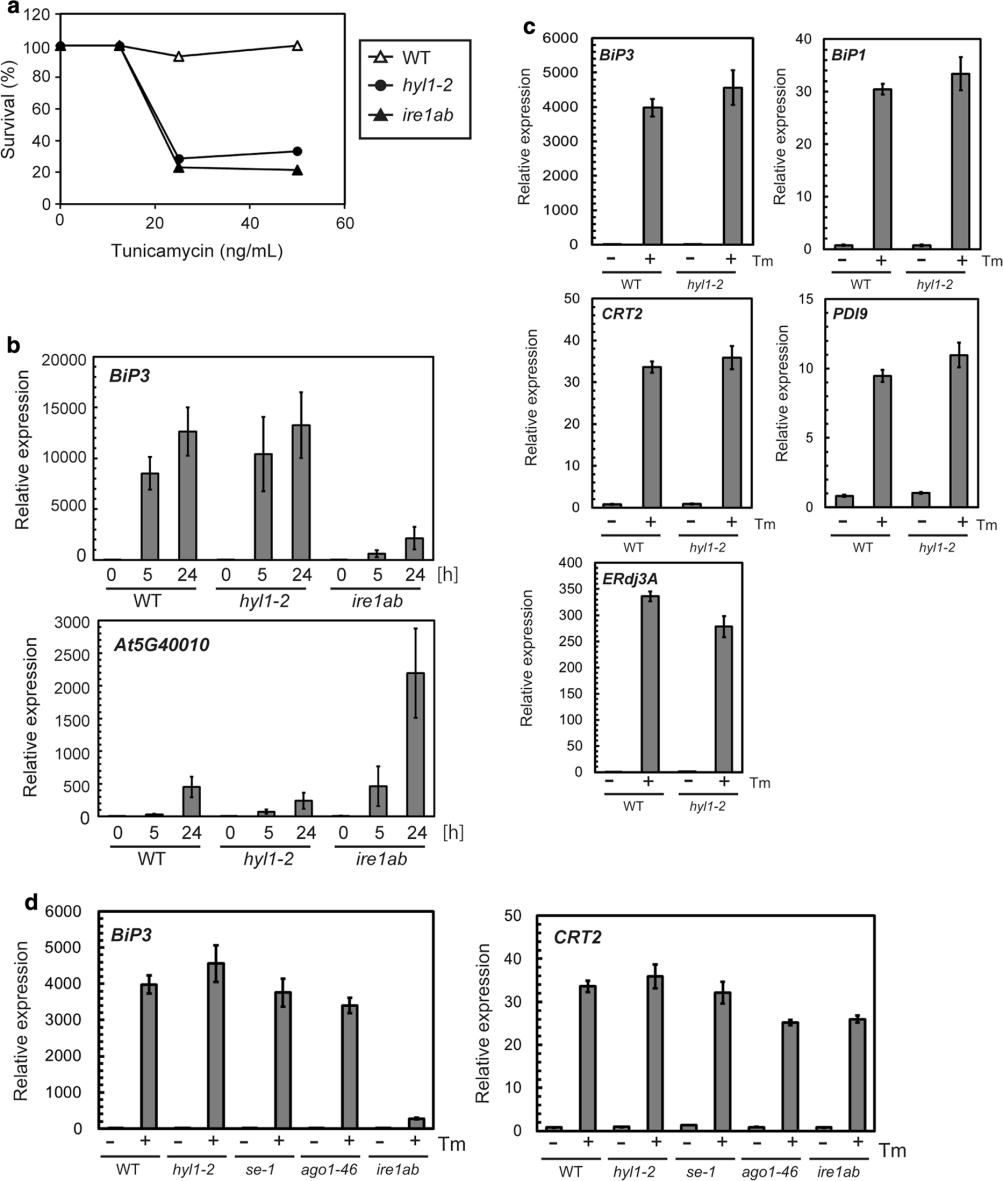
Sensitivity and transcriptional response of *hyl1-2* and *ire1ab* mutants to tunicamycin. **a** Effect of tunicamycin on survival of wild-type, *hyl1-2*, and *ire1ab*. Seeds of wild-type (WT), *hyl1-2*, and *ire1ab* were sown on half-strength MS medium with indicated concentrations of tunicamycin and grown for 10 days. The percentage of seeds that survived was calculated. **b** qRT-PCR analysis of wild-type and *hyl1-2* and *ire1ab* mutant seedlings to tunicamycin. Ten-day-old wild-type and *hyl1-2* and *ire1ab* mutant seedlings were treated with 5 μg/mL tunicamycin for indicated times and subjected to qRT-PCR analysis for detecting *BiP3* and *At5g40010*. The expression values of indicated genes were normalized to that of *Act8*. **c** qRT-PCR analysis of wild-type and *hyl1-2* mutant seedlings to tunicamycin. Ten-day-old wild-type and *hyl1-2* mutant seedlings were treated with 5 μg/mL tunicamycin (+Tm) or 0.1% DMSO (−Tm) as a solvent control for 5 h, and subjected to qRT-PCR analysis for detecting indicated genes. The expression values of indicated genes were normalized to that of *Act8*. **d** qRT-PCR analysis of wild-type and *hyl1-2*, *se-1*, and *ago1-46* mutant seedlings to tunicamycin. Ten-day-old wild-type and mutant seedlings were treated with tunicamycin or DMSO and subjected to qRT-PCR analysis for *BiP3* and *CRT2* as in **c**

We next compared the sensitivity of *hyl1-2* mutant to that of *ire1ab*, a known mutant that exhibit hypersensitivity to ER stress due to the defective transcriptional response to ER stress [11]. As shown in Fig. 2a, the hypersensitivity of *hyl1-2* mutant to tunicamycin was comparable to that of *ire1ab* mutant.

We then asked whether *hyl1-2* mutant is defective in the UPR. We treated wild-type and *hyl1-2* and *ire1ab* mutant seedlings with tunicamycin and subjected them to qRT-PCR analysis. We first detected *BiP3* as a gene induced in an IRE1-dependent manner in the early phase of the UPR [19, 20]. As shown in Fig. 2b, *BiP3* was upregulated in *hyl1-2* mutant as well as in wild-type seedlings, whereas *ire1ab* mutant seedlings showed compromised *BiP3* induction as previously reported [11]. We next detected another ER stress-responsive gene, *At5g40010*. *At5g40010* is a gene up-regulated to some extent in the wild-type but more strongly in *ire1ab* mutant, presumably because *ire1ab* mutant undergoes more severe cell damage [13]. As shown in Fig. 2b, *At5g40010* transcripts responded similarly to tunicamycin in *hyl1-2* mutant as well as in the wild-type, whereas *ire1ab* mutant exhibited stronger *At5g40010* induction as previously reported [13]. Other genes that encode ER-resident molecular chaperones and folding enzymes also exhibit similar expression both in the wild-type and *hyl1-2* mutant plants (Fig. 2c). We also tested *se-1* and *ago1-46* mutants, which exhibit similar tunicamycin sensitivity to the wild-type plants, for two ER chaperone genes; IRE1-dependent *BiP3* and IRE1-independent *CRT2*. As shown in Fig. 2d, induction of these two genes in both *se-1* and *ago1-46* mutants was similar to that in the wild-type (Fig. 2d). Taken together, the transcriptional response of *hyl1*-2, *se-1*, and *ago1-46* mutant plants is indistinguishable from that of wild-type plants.

### Discussion

We demonstrated in the present study that, among Arabidopsis miRNA-related mutants tested, only *hyl1-2* mutant was more sensitive to the ER stress inducer tunicamycin while *se-1* and *ago1-46* mutants did not exhibit such hypersensitivity. It suggests that an overall reduction in miRNA biogenesis and function is not the cause of the observed tunicamycin oversensitivity.

One possible interpretation is that miRNAs that show less accumulation specifically in *hyl1-2* mutant accounts for the observed hypersensitivity. Indeed, not all miRNAs are similarly affected among miRNA-related mutants [21], and the observable growth and morphological phenotypes of those mutants were not identical. Therefore, it is plausible that less accumulation of some of miRNAs whose accumulation is more dependent on HYL1 stabilizes their target mRNAs, resulting in ER stress sensitivity.

Another possible interpretation is that HYL1-specific function contributes to ER stress tolerance. In plants, miRNA biogenesis, in which pri-miRNA transcripts are processed by DCL1 to generate mature miRNA, occurs in the subnuclear bodies called D-bodies in the nucleus. HYL1 is a dsRNA-binding protein that increases pri-miRNA processing by DCL1 [1]. Although HYL1 is primarily localized in D-bodies and nucleoplasm in the nucleus, a subfraction of HYL1 is also detected in the cytoplasm [22]. Therefore, it is conceivable that cytoplasmically localized HYL1 plays a role in recovering cellular damages caused by ER stress.

There have been recent reports that implicate a link between miRNA function and the ER. It has been reported that AGO1 is a peripheral ER membrane protein and that miRNA-mediated translational repression occurs on the ER membrane in plants [23]. Furthermore, in animal systems, IRE1 has been reported to destabilize a subset of pre-miRNAs to reduce accumulation of select miRNAs and initiate apoptosis, demonstrating the involvement of the UPR signaling pathway component in miRNA biogenesis [24]. Those reports prompted us to speculate a possible connection between miRNA biogenesis and function machineries and the ER stress response. However, the present study was unable to identify such connections, because *hyl1-2* mutant exhibited a normal transcriptional response to ER stress despite its significantly less tolerance. Nevertheless, this study presents an interesting observation that one of miRNA biogenesis-related mutants exhibits hypersensitivity to ER stress-inducing agents. Further analyses such as genome-wide transcript profiling would be required to elucidate the role of HYL1 in ER stress tolerance and the UPR.

## Limitations

The shortcoming of this paper is that the function of HYL1 during the ER stress response remains to be elucidated due to the lack of further experimental analyses such as transcriptome profiling using RNA-seq.

## Supplementary information

**Additional file 1:** Primers used for qRT-PCR.

## Data Availability

The data obtained and analysed during the current study are available from the corresponding author on reasonable request.

## Abbreviations

AGO1: ARGONAUTE1
BiP: BINDING PROTEIN
DCL1: DICER-LIKE1
DMSO: dimethyl sulfoxide
dsRNA: double-stranded RNA
ER: endoplasmic reticulum
HYL1: HYPONASTIC LEAVES1
miRNA: microRNA
MS: Murashige and Skoog
pri-miRNA: primary miRNA
qRT-PCR: quantitative reverse transcription PCR
SE: SERRATE
UPR: unfolded protein response
