# Supplementary material for: Deficiency in the double-stranded RNA binding protein HYPONASTIC LEAVES1 increases sensitivity to the endoplasmic reticulum stress inducer tunicamycin in Arabidopsis
Source: BMC Res Notes. 2019 Sep 14;12:580. doi: 10.1186/s13104-019-4623-3 (PMC6744651; doi:10.1186/s13104-019-4623-3)
Supplement: Supplementary file 1 — Additional file 1: Primers used for qRT-PCR. [file 13104_2019_4623_MOESM1_ESM.docx]

**Additional file 1: Primers used for qRT-PCR.**

| Name | Nucleotide sequence (5’ – 3’) |
| --- | --- |
| BiP3-F | CGAAACGTCTGATTGGAAGAA |
| BiP3-R | GGCTTCCCATCTTTGTTCAC |
| At5g40010-F | ATTTGTTTGAAGCGATTGATTG |
| At5g40010-R | CCTCCTCTTCGCTTCCTCTT |
| Act8-F | TCAGCACTTTCCAGCAGATG |
| Act8-R | ATGCCTGGACCTGCTTCAT |
| CRT2-F | TTTCGAGGAGCGCTTTG |
| CRT2-R | CACTCCCCAGCAGTGTTATC |
| BiP1-F | TCAGTCCTGAGGAGATTAGTGCT |
| BiP1-R | TGCCTTTGAGCATCATTGAA |
| ERdj3A-F | CATCTGGCGGGTTCTAAG |
| ERdj3A-R | GCCGGAAACTTGTCTACC |
| PDI9-F | TGCATCTGTGGAACTGAACGC |
| PDI9-R | TTTTTGCAGCCCTCTTCCACTC |
